# Trichostatin A Influences Dendritic Cells’ Functions by Regulating Glucose and Lipid Metabolism via PKM2

**DOI:** 10.3390/molecules31020319

**Published:** 2026-01-16

**Authors:** Xiaoyu Yang, Lihui Men, Yan Guo, Linnan Duan, Meiyi Yu, Leyi Zhang, Tongtong Song, Xiang Li, Xia Chen

**Affiliations:** Department of Pharmacology, College of Basic Medical Science, Jilin University, Changchun 130021, China; yangxiaoyu20@mails.jlu.edu.cn (X.Y.); menlh@jlu.edu.cn (L.M.); yany.guo@outlook.com (Y.G.); duanln1997@163.com (L.D.); 17860554155@163.com (M.Y.); ly_louise@sina.com (L.Z.)

**Keywords:** trichostatin A, pyruvate kinase M2, dendritic cells, metabolism

## Abstract

Dendritic cells (DCs) play a crucial role in immune protection against myocardial infarction (MI). Through multiple experimental methods including bioinformatics, qPCR, Western blotting, immunofluorescence, MTT assays, echocardiography, TTC staining, and flow cytometry, this study found that metabolism was demonstrated to be markedly altered under oxygen–glucose deprivation (OGD) conditions in DCs. Pyruvate kinase M2 (PKM2) is a key protein in metabolism, and PKM2 was upregulated under OGD conditions in DCs. Trichostatin A (TSA) alleviated the OGD-induced cellular damage in DCs. Furthermore, TSA was shown to modulate DCs’ function by enhancing glycolysis while suppressing fatty acid synthesis and oxidation pathways. The metabolic changes caused by TSA and OGD were mechanistically mediated by PKM2. Mechanistically, PKM2 modulates glucose and lipid metabolism via its dimer formation. These results deepen our understanding of the interplay among TSA, glucose and lipid metabolism and DC functions in MI.

## 1. Introduction

Myocardial infarction (MI) remains a leading cause of mortality and disability worldwide, which causes more than 2.4 million deaths in the USA [1]. Thus, identifying efficient protective factors and elucidating underlying molecular mechanisms are critical for advancing MI treatment. Immune cells play a pivotal role in all stages of MI [2]. MI reduces dendritic cell (DC) infiltration in infarcted myocardial tissue, which is associated with increased macrophage infiltration, impaired reparative fibrosis and elevated cardiac rupture risk [3]. In addition, DC-mediated modulation of monocyte/macrophage homeostasis has been found to be involved in the post-MI healing process [4]. Trichostatin A (TSA), a histone deacetylase inhibitor, can interfere with DCs’ maturation, especially with the induction or suppression of cytokine production, and thus impact immune responses. IFNγ production decreased with the treatment of epigenetic agents [5]. In hepatic ischemia, DCs modulate macrophage phenotypic changes through the prostaglandin E receptor 3 signaling pathway [6]. These findings indicate that DCs may participate in cardiac protection in the infarcted heart.

Metabolism can regulate the functions of immune cells. Altering immune cell metabolism has emerged as a key subject in the immunological field [7]. In DCs, fatty acid oxidation modulation has shown therapeutic potential for type I hypersensitivity reactions, and glycolysis plays a role in antiviral immunity regulation against viral infection [8,9]. Pyruvate kinase M2 (PKM2) has been reported to regulate metabolism homeostasis in cancer cells and is involved in immune metabolism in macrophages [10,11,12,13]. PKM2 mainly exists in the forms of dimer and tetramer. The tetrameric form has high glycolytic activity, and is located in to the cytoplasm. The dimeric form localizes to the nucleus with low glycolytic activity and some gene regulatory roles, such as β-catenin, rG4, and STAT3, may be observed in nuclear translocation [14,15,16,17].

Previous studies have demonstrated that histone deacetylases (HDACs) deacetylate PKM2, increasing nuclear localization to promote glycolysis and regulate cell differentiation [18,19]. This finding indicates that HDACs may regulate PKM2 to change its functional properties. Notably, nuclear PKM2 has been shown to act as a transcriptional cofactor. The objective of the present study was to investigate the effect of TSA on DC damage under OGD conditions, and to understand how PKM2 regulates glucose and lipid metabolism, affecting DC injury from a metabolic standpoint.

## 2. Results

### 2.1. Changes in Glucose and Lipid Metabolism Occur in DCs Under Hypoxic Conditions

**Figure 1:**
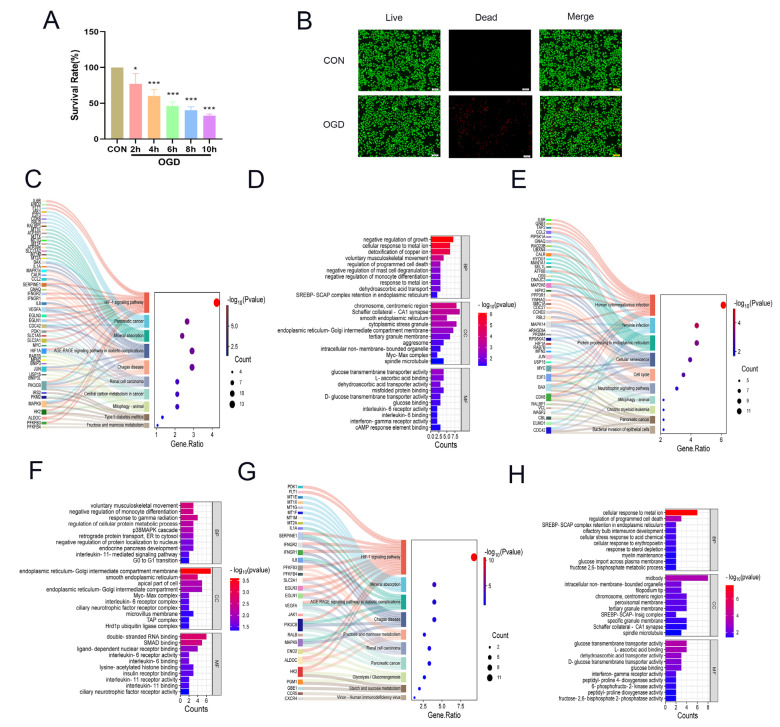
Changes in glucose and lipid metabolism occurred in DCs under hypoxic conditions. (**A**) OGD progressively inhibited the survival of DCs (*n* = 3). (**B**) Immunofluorescence analysis was performed to assess the survival of DCs after 4 h of OGD stress (green: calcein AM-stained live cells; red: propidium iodide-labeled dead cells) (*n* = 3). (**C**,**D**) KEGG and GO enrichment of the differentially expressed genes in the normoxia–nonsilenced–unstimulated DC and hypoxia–nonsilenced–unstimulated DC groups. (**E**,**F**) KEGG and GO enrichment analyses of the up-regulated differentially expressed genes between normoxia–nonsilenced–unstimulated and hypoxia–nonsilenced–unstimulated groups. (**G**,**H**) KEGG and GO enrichment analyses of the down-regulated differentially expressed genes between normoxia–nonsilenced–unstimulated and hypoxia–nonsilenced–unstimulated groups. Compared with the control group, * *p* < 0.05, *** *p* < 0.001.

To investigate the changes in DCs during MI, DCs were subjected to OGD conditions, and their survival rate at different time points was assessed. We found that OGD4h significantly reduced cell viability. To ensure successful model establishment and sufficient cell quantities for experiments, we selected 4 h OGD treatment as the optimal modelling duration (Figure 1A). The immunofluorescence results also demonstrated that OGD reduced the survival rate of DCs (Figure 1B). Altogether, the viability of DCs decreased under OGD conditions.

During MI, due to coronary artery occlusion, myocardial cells are ischemic and hypoxic, leading to myocardial cell damage and necrosis. To evaluate the physiological changes in DCs after MI, the hypoxia-non-silenced-unstimulated DC and normoxia-non-silenced-unstimulated DC groups in the GSE60729 dataset were analyzed. The Benjamini–Hochberg (BH) method was used for multiple-testing correction of the data. Then, the criteria for screening differentially expressed genes were set as adjusted *p* value < 0.05 and |log_2_FC| > 1. For Gene Ontology (GO) and Kyoto Encyclopedia of Genes and Genomes (KEGG) enrichment analyses of differentially expressed genes, we adopted the Benjamini–Hochberg method for multiple-testing correction, with adjusted *p* value (FDR) < 0.05 as the significance threshold. KEGG analysis demonstrated gene enrichment in HIF-1 signaling pathways and metabolism-related signaling pathways, including fructose and mannose metabolism and central carbon metabolism. GO analysis highlighted enrichment in the signaling pathway related to SREBP-SCAP complex retention in the endoplasmic reticulum (Figure 1C,D). These findings indicate a strong connection between hypoxia and metabolism. The roles of immune metabolism in functional regulation in immune cells, and whether metabolism is involved in DC regulation, were subsequently evaluated. KEGG analysis revealed that the central carbon metabolism was enriched in the upregulated genes (Figure 1E,F). In addition, fructose and mannose metabolism, starch and sucrose metabolism, glycolysis and central carbon metabolism were enriched in the downregulated genes. GO enrichment results revealed that down-regulated genes were enriched in the signaling pathways related to the sterol regulatory element-binding protein (SREBP)–SREBP cleavage-activating protein (SCAP)–insulin-induced gene 1 (Insig) complex and the cellular response to sterol depletion, activation of 6-phosphofructose-2-kinase, and activation of fructose-2,6-bisphosphate 2-phosphatase (PFKFB3) (Figure 1G,H). These findings collectively indicate that hypoxia predominantly affects the gene expression of metabolic pathways related to glucose and lipid metabolism. In conclusion, hypoxic conditions notably affect glucose and lipid metabolism in DCs.

### 2.2. Oxygen–Glucose Deprivation Affects Glucose and Lipid Metabolism and Upregulates PKM2 Expression

**Figure 2:**
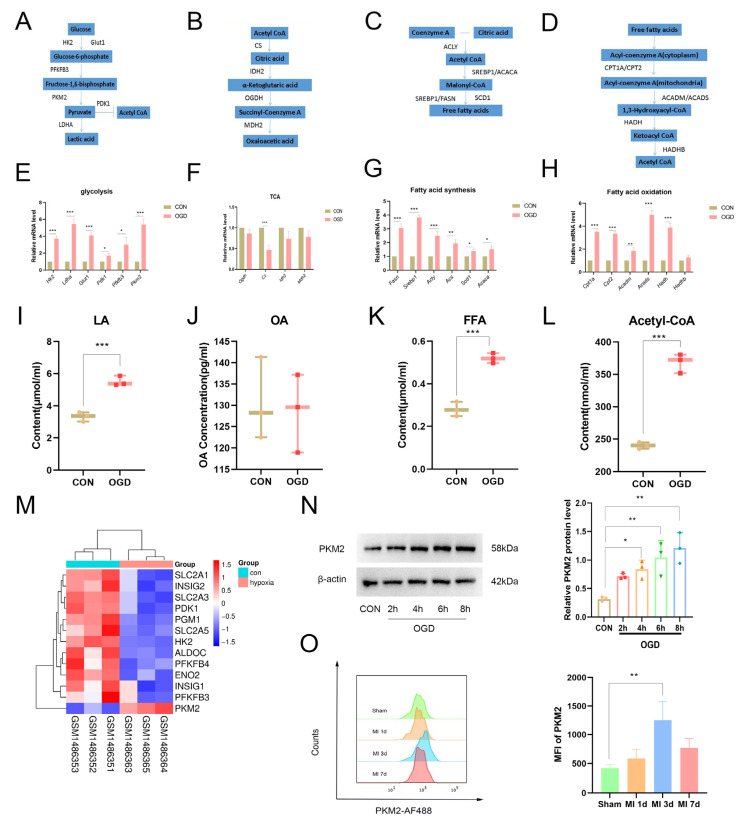
Oxygen–glucose deprivation affects glucose and lipid metabolism and upregulates PKM2 expression. (**A**) The key glycolytic metabolic pathway and the critical enzymes regulating the pathway. (**B**) The key TCA cycle pathway and the critical enzymes regulating the pathway. (**C**) The key fatty acid synthesis and the critical enzymes regulating the pathway. (**D**) The key fatty acid oxidation pathway and the critical enzymes regulating the pathway. (**E**) Transcriptional levels of glycolysis-related genes (*Hk2*, *Ldha*, *Glut1*, *Pdk1*, *Pfkfb3*, and *Pkm2*). (**F**) Expression of tricarboxylic acid cycle-related genes (*Ogdh*, *Cs*, *Idh2*, and *Mdh2*) was evaluated by RT-qPCR. (**G**) Fatty acid synthesis-related genes (*Fasn*, *Srebp1*, *Acly*, *Acs*, *Scd1*, and *Acaca*) were assessed by RT-qPCR. (**H**) Fatty acid oxidation-related genes (*Cpt1a*, *Cpt2*, *Acadm*, *Acads*, *Hadh*, and *Hadhb*) were analyzed using RT-qPCR. (**I**–**L**) Metabolic end-products were quantified. Glycolytic metabolism (lactate), tricarboxylic acid cycle metabolism (OA), fatty acid synthesis metabolism (FFA), and fatty acid oxidation metabolism (acetyl-CoA). (**M**) PKM2 was uniquely up-regulated in the GSE60729 dataset glycolysis-related gene heat map. (**N**) PKM2 is upregulated under OGD conditions in DCs. (**O**) PKM2 is upregulated in DCs in infarcted tissues. Compared with control group, * *p* < 0.05, ** *p* < 0.01, *** *p* < 0.001 (*n* = 3).

The aforementioned bioinformatics analysis results showed that glucose and lipid metabolism in hypoxia-induced DCs underwent significant changes. To investigate the alterations in glucose and lipid metabolism in DCs under OGD conditions, we selected key enzymes from the glycolytic (Figure 2A), TCA cycle (Figure 2B), fatty acid synthesis (Figure 2C), and fatty acid oxidation (Figure 2D) pathways and assessed their expression via qPCR. The results demonstrated that OGD promoted glycolysis (Figure 2E), fatty acid synthesis (Figure 2G), and oxidation (Figure 2H) pathways but had no effect on the tricarboxylic acid (TCA) cycle except CS (Figure 2F). Furthermore, the changes in the final metabolites of glucose–lipid metabolism were evaluated, which were consistent with the qPCR results (Figure 2I–L).

Analysis of the GSE60729 dataset revealed that genes related to glucose and lipid metabolism regulation were all downregulated, while PKM2 was uniquely upregulated (Figure 2M), indicating that PKM2 may have extra biological functions in addition to glycolysis. Therefore, the expression of PKM2 under OGD conditions was assessed at different time points: 0, 2, 4, 6, and 8 h (Figure 2N). The results showed that PKM2 began to increase at 4 h, and its expression levels continued to rise as the duration of OGD was prolonged in DCs. Additionally, the expression levels of PKM2 in DCs were assessed in infarcted tissues from mice at 1, 3, and 7 days following MI (Figure 2O). The results demonstrated that PKM2 expression in DCs in infarcted tissues reached its peak at 3 days post-MI. This suggests that PKM2 may have a function in DCs under OGD conditions.

### 2.3. TSA Ameliorates DC Injury

**Figure 3:**
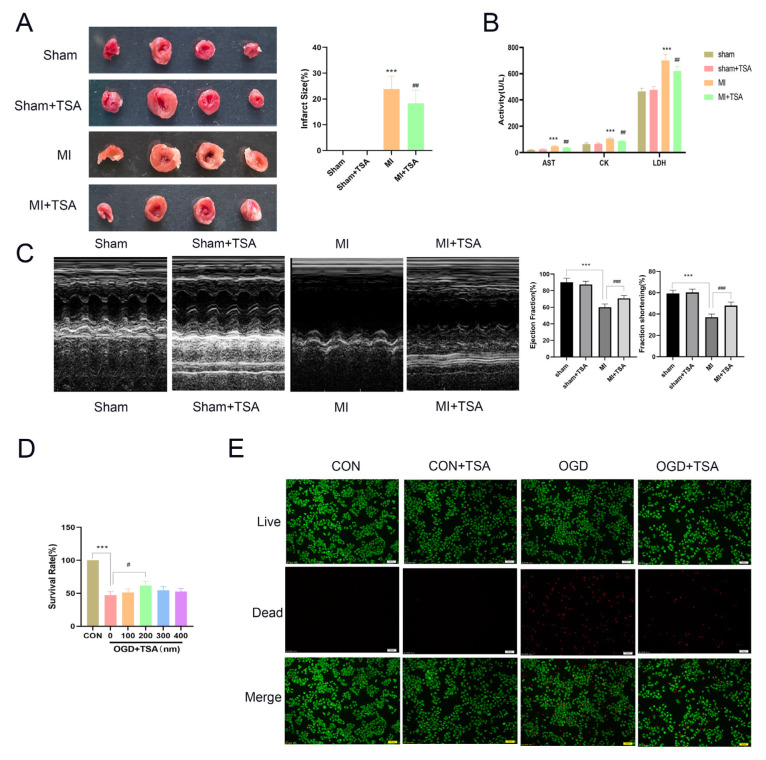
TSA ameliorates oxygen–glucose deprivation-induced injury in DCs. (**A**) TTC staining demonstrating the myocardial infarction area (*n* = 10). (**B**) Serum enzyme levels (AST, CK, and LDH) in mouse serum (*n* = 10). (**C**) Echocardiography reflects myocardial infarction status (*n* = 10). (**D**) Dose-dependent protective effects of TSA on OGD-induced DC injury evaluated by MTT assay (*n* = 3). (**E**) Impact of TSA on DC2.4 survival rate (green: calcein AM-stained live cells; red: propidium iodide-labeled dead cells) (*n* = 3). Compared with sham group and control group, *** *p* < 0.001; compared with MI group and OGD group, ^#^ *p* < 0.05, ^##^ *p* < 0.01, ^###^ *p* < 0.001.

The aforementioned results indicate that OGD promotes glycolysis, fatty acid synthesis and oxidation in DCs, and OGD promotes the expression of PKM2. Our previous research indicated that TSA can alleviate the symptoms of myocardial infarction [20]. However, the mechanism by which TSA mitigates damage to dendritic cells in the heart remains unclear. To investigate this, we conducted the following experiments. An MI model was established by ligating the left anterior descending branch of the coronary artery to investigate the effect of TSA on MI in 3–5-week-old mice. The results demonstrated that TSA significantly reduced the infarct area in mice with MI (Figure 3A). Serum enzymes, including AST, CK, and LDH, can reflect the severity of MI to some extent. Following MI, the levels of AST, CK and LDH in mice increased; however, after the administration of TSA, these cardiac enzyme levels decreased (Figure 3B). The echocardiography results showed decreased EF and FC values during MI, which increased after TSA administration (Figure 3C). These findings suggest that TSA exerts a protective effect against MI.

Additionally, previous research has demonstrated that TSA enhances the infiltration of DCs in rat MI tissue, and that DCs-derived exosomes improve post-MI cardiac function while alleviating associated symptoms [21]. Based on the aforementioned results, we hypothesized that TSA may directly protect DCs and indirectly enhance the anti-MI capacity. Consequently, to investigate TSA protective effects, its ability to sustain DCs’ survival under OGD conditions, which simulate MI, was evaluated. MTT results indicate that TSA significantly promotes DC survival under OGD conditions at 200 nm. Therefore, we selected 200 nM for subsequent experiments (Figure 3D). DCs’ cellular viability under OGD stress treated with or without TSA was quantitatively assessed by employing an immunofluorescence assay; live cells were labeled with calcein AM, while dead cells were labeled with PI (Figure 3E). The findings indicate that TSA significantly mitigated OGD-induced cell death compared with untreated controls in DCs. These findings demonstrate that TSA mitigates OGD-induced DC injury.

### 2.4. TSA Promotes Glycolysis While Suppressing Fatty Acid Synthesis and Oxidation Under OGD Conditions in DCs

**Figure 4:**
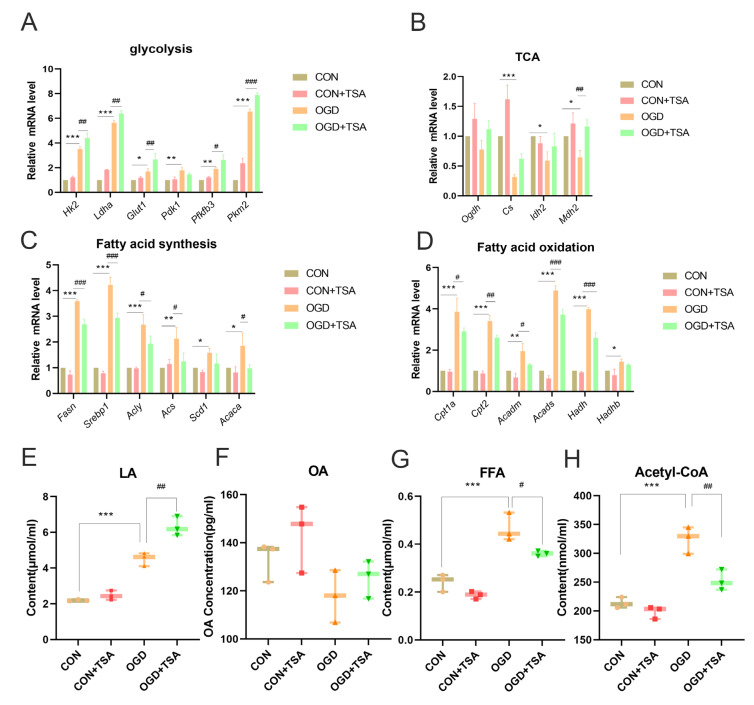
Effects of TSA on glucose and lipid metabolism in DC2.4 cells. The experiment was conducted across four groups: CON (DC2.4 cells cultured for 4 h under normal conditions), CON + TSA (DC2.4 cells cultured for 4 h with 200 nM TSA), OGD (DC2.4 cells cultured for 4 h under OGD conditions), and OGD + TSA (DC2.4 cells cultured for 4 h with 200 nM TSA under OGD conditions). (**A**) Transcriptional levels of glycolysis-related genes (*Hk2*, *Ldha*, *Glut1*, *Pdk1*, *Pfkfb3*, *and Pkm2*) (*n* = 3). (**B**) Expression of tricarboxylic acid cycle-related genes (*Ogdh*, *Cs*, *Idh2*, *and Mdh2*) was evaluated by RT-qPCR (*n* = 3). (**C**) Fatty acid synthesis-related genes (*Fasn*, *Srebp1*, *Acly*, *Acs*, *Scd1*, and *Acaca*) were assessed by RT-qPCR (*n* = 3). (**D**) Fatty acid oxidation-related genes (*Cpt1a*, *Cpt2*, *Acadm*, *Acads*, *Hadh*, and *Hadhb*) were analyzed using RT-qPCR (*n* = 3). (**E**–**H**) Metabolic end-products were quantified. Glycolytic metabolism (lactate), tricarboxylic acid cycle metabolism (OA), fatty acid synthesis metabolism (FFA), and fatty acid oxidation metabolism (acetyl-CoA) (*n* = 3). Compared with control group, * *p* < 0.05, ** *p* < 0.01, *** *p* < 0.001; compared with OGD group, ^#^ *p* < 0.05, ^##^ *p* < 0.01, ^###^ *p* < 0.001.

Due to the established role of TSA and hypoxia-induced changes in DCs, glucose and lipid metabolism in DCs was subsequently evaluated under OGD conditions with TSA treatment using RT-qPCR. First, the mRNA expression of metabolic enzymes in key pathways regulating glucose and lipid metabolism was assessed. In DC2.4 cells, transcriptional levels of glycolysis-related genes [Hexokinase 2 (*Hk2*), *Ldha*, Glucose transporter 1 (*Glut1*), Pyruvate dehydrogenase kinase 1 (*Pdk1*), *Pfkfb3*, and *Pkm2*] were upregulated under OGD conditions. Notably, TSA could further enhance transcription of glycolysis-related genes (*Hk2*, *Ldha*, *Glut1*, *Pfkfb3*, and *Pkm2*) compared with the OGD group (Figure 4A).

In addition, the key enzymes in the TCA cycle metabolic pathway were analyzed. The transcriptional levels of TCA cycle-related genes [*Citrate synthase* (*Cs*), *Isocitrate dehydrogenase (NADP(+)) 2* (*Idh2*), and *Malate dehydrogenase 2* (*Mdh2*)] were downregulated under OGD conditions. Compared with the OGD group, TSA increased *Mdh2* expression but showed no significant effect on *Cs*, *Idh2*, and *Ogdh* (Figure 4B). The effect of TSA on fatty acid metabolism was subsequently assessed in DC2.4 cells. Transcriptional levels of fatty acid synthesis-related and fatty acid oxidation-related genes were evaluated, and fatty acid synthesis-related genes [*Fatty acid synthase* (*Fasn*), *Srebp1*, *ATP citrate lyase* (*Acly*), *Acetyl-CoA synthetase* (*Acs*), and *Acetyl-CoA carboxylase α* (*Acaca*)] and fatty acid oxidation-related genes [*Carnitine palmitoyltransferase 1A* (*Cpt1a*), *Cpt2*, *Acyl-CoA dehydrogenase medium chain* (*Acadm*), *Acad short chain* (*Acads*), *Hydroxyacyl-CoA dehydrogenase* (*Hadh*), and *Hydroxyacyl-CoA dehydrogenase Trifunctional multienzyme complex subunit β* (*Hadhb*)] were elevated under OGD conditions. Conversely, TSA treatment reversed this trend and downregulated the transcriptional levels of fatty acid synthesis-related genes (*Fasn*, *Srebp1*, *Acly*, *Acs*, and *Acaca*) and fatty acid oxidation-related genes (*Cpt1a*, *Cpt2*, *Acadm*, *Acads*, and *Hadh)* compared with the OGD group (Figure 4C,D). Therefore, in DC2.4 cells, OGD enhances the expression of genes associated with glycolysis, fatty acid synthesis, and fatty acid oxidation, whereas TSA amplifies the OGD-induced glycolytic response but suppresses both fatty acid synthesis and oxidation pathways after OGD exposure.

The metabolic end-products were further assessed: lactate (glycolytic metabolism), oxaloacetic acid (TCA cycle metabolism), fatty acid (fatty acid synthesis metabolism), and acetyl coenzyme A (fatty acid oxidation metabolism). DC2.4 cells exhibited increased biosynthesis of lactate, fatty acids, and acetyl coenzyme A under OGD treatment. Notably, TSA treatment promoted lactate biosynthesis but suppressed fatty acid and acetyl coenzyme A biosynthesis in DC2.4 cells (Figure 4E–H). Collectively, these data indicate that TSA regulates glucose and lipid metabolism in DCs following OGD treatment.

### 2.5. TSA Modulates Glucose and Lipid Metabolism via PKM2 in DCs Under OGD Conditions

**Figure 5:**
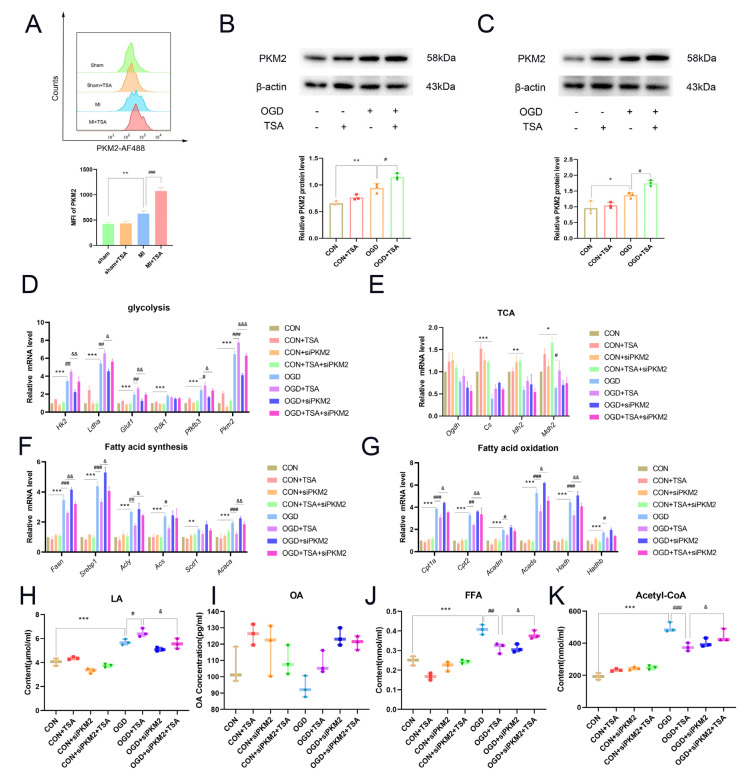
The effect of PKM2 interference on TSA regulates glucose and lipid metabolism in DCs after OGD treatment. (**A**) Flow cytometry detection of PKM2 expression in dendritic cells from mouse myocardial infarction tissue (*n* = 3). (**B**,**C**) TSA affects PKM2 expression in DCs (*n* = 3). (**D**) The mRNA levels of glycolysis-related genes (*Hk2*, *Ldha*, *Glut1*, *Pdk1*, *Pfkfb3*, and *Pkm2*) detected by RT-qPCR. The experiments were divided into eight groups: CON (DC2.4 cells cultured for 4 h), CON + TSA (200 nM TSA treatment for 4 h), CON + si-PKM2 (siRNA transfection for 48 h, followed by 4 h culture), CON + si-PKM2 + TSA (siRNA transfection for 48 h, followed by 200 nM TSA treatment for 4 h), OGD (cultured under OGD conditions for 4 h), OGD + TSA (cultured under OGD conditions with 200 nM TSA treatment for 4 h), OGD + si-PKM2 (siRNA transfection for 48 h, followed by 4 h culture under OGD conditions), and OGD + si-PKM2 + TSA (siRNA transfection for 48 h, followed by treatment with 200 nM TSA for 4 h under OGD conditions). The following (**E**–**K**) are grouped in the same abbreviation (*n* = 3). (**E**) The mRNA levels of TCA-related genes (*Ogdh*, *Cs*, *Idh2*, and *Mdh2*) were detected by RT-qPCR (*n* = 3). (**F**) The mRNA levels of fatty acid synthesis-related genes (*Fasn*, *Srebp1*, *Acly*, *Acs*, *Scd1*, and *Acaca*) were detected by RT-qPCR (*n* = 3). (**G**) The mRNA levels of fatty acid oxidation-related genes (*Cpt1a*, *Cpt2*, *Acadm*, *Acads*, *Hadh*, and *Hadhb*) were detected by RT-qPCR (*n* = 3). (**H**–**K**) Levels of lactate, OA, FFA, and acetyl-CoA in DC2.4 cells (*n* = 3). Compared with sham group and control group, * *p* < 0.05, ** *p* < 0.01, *** *p* < 0.001; compared with MI group and OGD group, ^#^ *p* < 0.05, ^##^ *p* < 0.01, ^###^ *p* < 0.001; compared with OGD + TSA group, ^&^ *p* < 0.05, ^&&^ *p* < 0.01, ^&&&^ *p* < 0.001.

To determine the specific mechanism by which TSA regulates glucose and lipid metabolism via PKM2, flow cytometry was used to assess the expression of PKM2 in DCs from the hearts of mice with MI. PKM2 was upregulated in DCs during MI and further upregulated after the addition of TSA (Figure 5A). Subsequently, PKM2 expression was assessed in DC2.4 cells and BMDCs. BMDCs were isolated from C57BL/6 mouse bone marrow and induced to mature (Figure S1). PKM2 was upregulated under OGD conditions and TSA further upregulated the expression of both DC2.4 cells and BMDCs (Figure 5B,C), indicating that PKM2 may have essential biological functions in DCs under OGD conditions and TSA upregulates PKM2 expression to exert its protective effects. To investigate the functional role of PKM2, si-PKM2 interference was performed. Si-PKM2 interference downregulated the mRNA levels of glycolysis-related genes (*Hk2*, *Ldha*, *Glut1*, *Pkm2*, and *Pfkfb3*), compared with the OGD + TSA group (Figure 5D), indicating TSA enhanced glycolytic metabolism via PKM2 after OGD treatment.

By contrast, si-PKM2 had no significant differences in TCA-related genes (Figure 5E). In DC2.4 cells, si-PKM2 interference upregulated fatty acid synthesis-related genes (*Fasn*, *Srebp1*, *Acly*, and *Acaca*) post-OGD compared with the TSA treatment group (Figure 5F). This suggests TSA inhibits fatty acid synthesis in DC2.4 cells via PKM2 upon OGD conditions. Similarly, si-PKM2 interference elevated fatty acid oxidation-related genes (*Cpt1a*, *Cpt2*, *Acads*, and *Hadh*) under OGD conditions compared with the TSA treatment group (Figure 5G), indicating TSA suppresses fatty acid oxidation metabolism via PKM2 under OGD conditions.

The effects of PKM2 on glucose and lipid end metabolites upon TSA treatment were evaluated. Si-PKM2 interference inhibited the biosynthesis of lactate but promoted the biosynthesis of free fatty acids and acetyl coenzyme A post-OGD compared with the OGD group (Figure 5H–K). Collectively, the aforementioned findings indicate that TSA enhances glycolysis while suppressing fatty acid synthesis and oxidation via PKM2 under OGD conditions.

### 2.6. TSA Regulates Glucose and Lipid Metabolism in DCs Through PKM2 Dimerization

**Figure 6:**
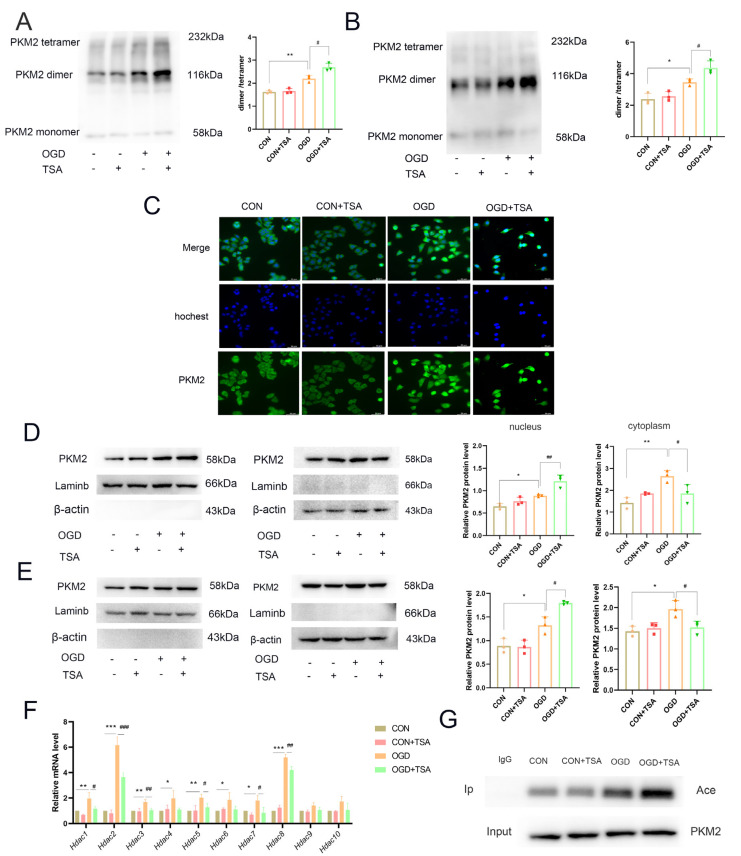
TSA promotes PKM2 dimer in DCs. (**A**) DSS experiments showed the changes in PKM2 in dimeric and tetrameric forms in DCs with TSA (*n* = 3). (**B**) DSS experiments showed the changes in nuclear PKM2 in dimeric and tetrameric forms in DCs (*n* = 3). (**C**) Subcellular localization and expression of PKM2 in DC2.4 cells were visualized by immunofluorescence (scale bar = 50 μm) (*n* = 3). (**D**) The nuclear and cytoplasmic protein expression level of PKM2 in DC2.4 cells was detected by Western blot (*n* = 3). (**E**) The nuclear and cytoplasmic protein expression level of PKM2 in BMDCs was detected by Western blot (*n* = 3). (**F**) The mRNA levels of *Hdac1–10* in DCs were detected by RT-qPCR (*n* = 3). (**G**) P*KM2* deacetylation in DCs (*n* = 3). Compared with control group, * *p* < 0.05, ** *p* < 0.01, *** *p* < 0.001; compared with OGD group, ^#^ *p* < 0.05, ^##^ *p* < 0.01, ^###^ *p* < 0.001.

The aforementioned studies indicate that TSA promotes glycolysis in DCs through PKM2, thereby inhibiting fatty acid synthesis and oxidation pathways. However, the specific mechanism by which PKM2 regulates glucose and lipid metabolism remains unknown. In mammalian cells, there exist three dynamic PKM2 forms: monomeric, dimeric and tetrameric PKM2. The dimeric form of PKM2 is known to regulate glycolysis and localize to the nucleus. Due to the functional importance of PKM2’s subcellular distribution and three forms, the alterations of the dimeric and tetrameric forms were evaluated using DSS experiments. The results showed that the PKM2 dimer increased under OGD conditions. TSA treatment increased the ratio of dimeric PKM2 compared with the OGD group (Figure 6A). Consequently, nuclear proteins were isolated from DCs for DSS cross-linking, and the results showed that TSA further promoted PKM2 expression in nuclear proteins (Figure 6B).

The subcellular localization of PKM2 was subsequently assessed using immunofluorescence in DC2.4 cells under OGD conditions with TSA treatment. PKM2 expression increased in the nucleus in OGD conditions and was further enhanced following TSA treatment (Figure 6C). Meanwhile, the distribution of PKM2 was observed in both the nucleus and cytoplasm. To confirm these findings, a nuclear-cytosol separation kit was employed to isolate nuclear and cytoplasmic proteins from DCs followed by Western blotting. Notably, compared with untreated controls, PKM2 expression was elevated in the nucleus following OGD treatment. Furthermore, TSA treatment further amplified nuclear PKM2 accumulation in DC2.4 cells and BMDCs (Figure 6D,E), suggesting that TSA promotes PKM2 nuclear translocation to exert its functional effects.

Research has reported, HDAC8 deacetylate PKM2 K62 to promote its nuclear translocation for glycolysis regulation [18], PKM2 K433 acetylation promotes nuclear translocation and protein kinase activity [22]. These studies indicated that PKM2 acetylation influences the equilibrium between PKM2 dimers and tetramers. TSA, a HDAC inhibitor, is known to affect HDAC I and IIa classes activity. Therefore, we hypothesize that TSA modulates PKM2 dimerization by influencing its acetylation status. Firstly, the potential regulatory targets of TSA (*Hdac1–10*) were assessed using RT-qPCR. OGD condition upregulated *Hdac1–8* expression, while TSA treatment specifically downregulated *Hdac1*, *2*, *3*, *5*, *7*, and *8*, with *Hdac2* showing the most pronounced suppression (Figure 6F). Then, we assessed the acetylation status of PKM2 via CO-IP acetylation experiment. The results demonstrated that TSA increased PKM2 acetylation level under OGD conditions (Figure 6G).

**Figure 7:**
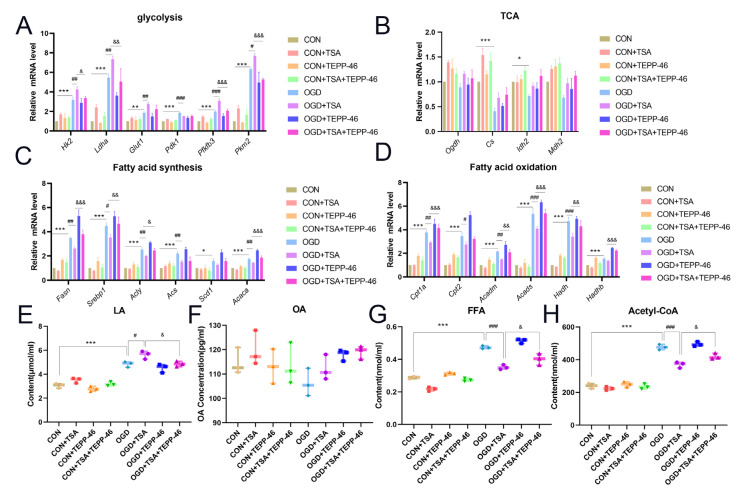
TSA regulates glucose and lipid metabolism in DCs after OGD treatment via PKM2 dimer. The experiments were divided into eight groups: CON (DC2.4 cells cultured for 4 h), CON + TSA (200 nM TSA treatment for 4 h), CON + TEPP-46 (50 μM TEPP-46 treatment for 1 h, followed by 4 h culture), CON + TEPP-46 + TSA (50 μM TEPP-46 treatment for 1 h, followed by 200 nM TSA treatment for 4 h), OGD (cultured under OGD conditions for 4 h), OGD + TSA (cultured with 200 nM TSA treatment for 4 h under OGD conditions), OGD + TEPP-46 (50 μM TEPP-46 treatment for 1 h, followed by 4 h culture under OGD conditions), and OGD + TEPP-46 + TSA (50 μM TEPP-46 treatment for 1 h, followed by treatment with 200 nM TSA for 4 h under OGD conditions). (**A**) The mRNA levels of glycolysis-related genes (*Hk2*, *Ldha*, *Glut1*, *Pdk1*, *Pfkfb3*, *and Pkm2*) detected by RT-qPCR (*n* = 3). (**B**) The mRNA levels of tricarboxylic acid cycle-related genes (*Ogdh*, *Cs*, *Idh2*, and *Mdh2*) detected by RT-qPCR (*n* = 3). (**C**) The mRNA levels of fatty acid synthesis-related genes (*Fasn*, *Srebp1*, *Acly*, *Acs*, *Scd1*, and *Acaca*) detected by RT-qPCR (*n* = 3). (**D**) The mRNA levels of fatty acid oxidation-related genes (*Cpt1a*, *Cpt2*, *Acadm*, *Acads*, *Hadh*, and *Hadhb*) detected by RT-qPCR (*n* = 3). (**E**–**H**) Levels of lactate, OA, FFA, and acetyl-CoA in DC2.4 cells (*n* = 3). Compared with control group, * *p* < 0.05, ** *p* < 0.01, *** *p* < 0.001; compared with OGD group, ^#^ *p* < 0.05, ^##^ *p* < 0.01, ^###^ *p* < 0.001; compared with OGD + TSA group, ^&^ *p* < 0.05, ^&&^ *p* < 0.01, ^&&&^ *p* < 0.001.

To further validate that TSA regulates glucose and lipid metabolism in DCs through PKM2 dimerization, we need to suppress PKM2 dimer and assess metabolic alterations in DCs. TEPP-46, acting as a PKM2 tetramer stabilizer, indirectly reduces its nuclear translocation levels. Therefore, we administered TEPP-46 to dendritic cells to evaluate alterations in glucose and lipid metabolism. The results demonstrated that, compared with the OGD + TSA group, after administration of TEPP-46, the glycolysis-related genes *Hk2*, *Ldha*, *Pfkfb3*, and *Pkm2* were upregulated (Figure 7A), while there were no significant changes in the TCA cycle-related genes (Figure 7B). The fatty acid synthesis-related genes *Fasn*, *Srebp1*, *Acly*, and *Acaca*, as well as the fatty acid oxidation-related genes *Cpt1a*, *Acadm*, *Acads*, and *Hadh*, were all upregulated (Figure 7C,D). These results indicate that TSA regulates glucose and lipid metabolism in DCs through PKM2 dimerization. Metabolic end products also showed the same results (Figure 7E–H). In summary, PKM2 dimerization exerts its regulatory role in glucose and lipid metabolism, regulated by TSA in DCs under OGD conditions.

## 3. Discussion

DCs have been found to exert a protective role in MI. DCs aggregate in the infarcted area via fibroblasts after MI and protect cardiomyocytes. Notably, cardiomyocytes release cytokines or metabolites to recruit additional immune cells against MI. There are previous studies showing treatment of cardiovascular diseases by targeting DCs [21,23,24]. Our prior research revealed that TSA promotes DC recruitment to the MI area in rats. In the present study, an OGD model was utilized to simulate MI and showed TSA ameliorated OGD-induced DC injury. These findings highlight the critical role of post-infarction DCs in mitigating cardiac damage.

It has been discovered that targeting immune cells’ metabolism can dynamically regulate their function for therapeutic purposes. The results showed that hypoxia caused significant alterations in metabolic pathways in DCs, particularly in glucose and lipid metabolism pathways. In addition, hypoxia has been reported to promote glycolysis in cardiomyocytes [25], immune cells [26] and fibroblasts [27], protecting cells from hypoxic damage. The present findings further demonstrated that TSA promotes DC glycolysis following OGD treatment.

It has been reported that the hypoxia–lactate axis can mitigate inflammation, and the glycolytic metabolite lactate directly suppresses signaling pathways and modifies histones, which play a pivotal role in regulating macrophage polarization, tumor immunity and antiviral responses [28]. Proper glycolysis promotes cell survival while attenuating inflammatory responses. Furthermore, hypoxia stimulates lipid biosynthesis and promotes lipid peroxidation [29,30]. Notably, extracellular vesicles derived from hypoxic mesenchymal stem cells improve renal fibrosis after ischemia–reperfusion injury by restoring CPT1A-mediated fatty acid oxidation [31]. Fatty acid β-oxidation is stimulated following MI to produce cardiac energy during ischemia [32]. While suppression of fatty acid synthesis has been reported to accelerate recovery from an ischemic stroke [33], the present study demonstrated that TSA inhibits both fatty acid and oxidation processes in OGD-exposed DCs. Collectively, the results indicate that altered fatty acid metabolic and glycolysis pathways may affect the progression of ischemic diseases.

PKM2 is a primary rate-limiting enzyme in the glycolysis pathway and a key regulator of immunometabolism [10], which has been implicated in multiple homeostasis processes. In amino acid metabolism, PKM2 enhances collagen synthesis and secretion in myofibroblasts by upregulating glycine metabolism [34]. Additionally, it promotes serine anabolism to sustain cancer cell proliferation [35]. In lipid metabolism, PKM2 interacts with sterol Srebps to modulate lipid dynamics [11] and coordinates the metabolic switch between glycolysis and glutaminolysis [13]. The present study revealed that TSA significantly upregulates PKM2 expression in DCs, highlighting its role in regulating the metabolic switch. Specifically, TSA enhances glycolysis, suppresses fatty acid metabolism and protects against OGD-induced DC injury via PKM2-dependent mechanisms.

Research indicates a close association between the nuclear translocation of PKM2 and glucose–lipid metabolism. In macrophages derived from both human and mouse fibrotic livers, FSTL1 directly binds to PKM2, promoting its phosphorylation and subsequent nuclear translocation. This process enhances PKM2-dependent glycolysis and strengthens M1 polarization. Inhibiting the nuclear translocation of PKM2 partially counteracts the glycolytic and inflammatory effects mediated by FSTL1 [36]. In colorectal cancer cells, DDX39B recruits importin α5, accelerating the nuclear translocation of PKM2 and promoting the expression of glycolysis-related genes [37]. Additionally, VB5 inhibits glycolysis and STAT3 phosphorylation by suppressing PKM2 phosphorylation and nuclear transport, facilitating metabolic reprogramming in Th17 cells [38]. These studies collectively demonstrate a significant relationship between PKM2 nuclear translocation and glycolysis. Yu Jutao et al. discovered that IGFBP7 binds to PKM2, promoting acetylation at the K433 site. This modification enhances PKM2 dimerization and nuclear translocation, subsequently accelerating lipid biogenesis and renal fibrosis through a SREBP1-dependent mechanism [39]. Reports indicate that protein kinase C epsilon (PKCε) interacts with PKM2, promoting its nuclear translocation and enhancing de novo lipid synthesis and tumor growth in prostate cancer cells [40]. In microglia, PKM2 dimerization promotes SREBP1 activation, driving lipid droplet formation and exacerbating mitochondrial dysfunction; inhibition of PKM2 dimerization reverses these effects [41]. These findings further illustrate the close relationship between PKM2 nuclear translocation and fatty acid metabolism. Our experimental results suggest that TSA may regulate glycolysis and fatty acid synthesis/oxidation pathways in dendritic cells through PKM2 dimerization.

TSA, a HDAC inhibitor, can inhibit class I and II HDACs. The research indicated that TSA can promote synapse elongation in DCs, thereby enhancing the antigen-presenting capacity of DCs. The mechanism involves inhibiting HDACs, promoting the Src family kinase/PI3K/Rho pathway, subsequently activating actin polymerization to facilitate synapse elongation [42]. Other studies have also found that TSA inhibits DCs’ maturation by downregulating the NF-κB pathway [43]. TSA reduces interferon secretion by DCs by inhibiting IFN regulatory factor 7 nuclear translocation [44]. TSA treatment of mouse DCs at non-apoptotic concentrations strongly inhibited TLR agonist-induced secretion of IL-1. In addition, TLR-mediated upregulation of co-stimulatory molecules was also inhibited [45]. In summary, TSA influences DC immune functions such as antigen presentation, maturation, and cytokine secretion, with mechanisms primarily related to inflammatory pathways. Our experimental results suggest that TSA may mitigate OGD-induced damage in DCs by regulating glucose and lipid metabolism.

The present results revealed that TSA primarily regulates PKM2 expression and further protects DCs from OGD conditions. HDAC8 regulates PKM2 by affecting its nuclear localization to promote glycolysis [18], and HDAC6 regulates the nuclear translocation of PKM2 to affecting the differentiation of Th17 cells [19]. HDAC3 affects the NF-κB/p-STAT3 signaling pathway by regulating PKM2, which in turn affects colon cancer progression [46]. In summary, the modulation of PKM2 acetylation can regulate its nuclear translocation, thereby influencing the control of glycolytic and lipid metabolism. Our findings indicate that TSA influences the ratio of PKM2 dimers to tetramers by affecting PKM2 acetylation, which subsequently impacts the regulation of glycolytic and lipid metabolism.

This study has several limitations. We cannot rule out the non-glycolytic sources of lactate and fail to reflect the dynamic changes within the glycolytic pathway. Therefore, the quantification of lactate alone cannot fully confirm that the lactate is entirely attributable to the glycolytic pathway. Nevertheless, in our experiments, we detected upregulated lactate which is the final metabolite of the glycolytic pathway. Lactic acid is a core metabolite in the glycolytic pathway. The increase in the level of lactate indicates that the overall output function of the glycolytic pathway was enhanced. Furthermore, we analyzed the changes in the key metabolic genes of the glycolytic pathway, which confirmed that the molecular regulation of this pathway was activated. Based on these, we conclude that the increased lactate level is mainly derived from the enhancement of the glycolytic pathway, and that the changes in lactate level can, to a certain extent, reflect the alterations in the glycolytic pathway. In addition, in vitro experiments revealed that TSA mitigates DC dysfunction by regulating glycolytic and lipid metabolism via PKM2, elucidating its underlying mechanism. Given the limitations of in vitro studies, we conducted further validation through animal experiments, thereby establishing a theoretical foundation for future clinical trials. Additionally, the direct target genes of PKM2 will be investigated. In siRNA knockdown experiments, although negative control (NC) siRNAs partially alleviate off-target effects, the theoretical risk of off-targeting cannot be fully abrogated. NCBI BLAST (BLAST+ 2.17.0) alignment of our designed siRNA sequences identified Ino80d and Ccdc30 as potential off-target genes. However, no studies have confirmed their association with PKM2 or metabolic pathways. Nevertheless, off-target effects remain a significant challenge for RNAi technology, and this limitation must be acknowledged. Where required, complementary techniques such as CRISPR-Cas9 gene knockout should be used to validate results, eliminate technical variability, and strengthen the rigor of conclusions. The siRNA-based findings in this study represent only preliminary evidence for PKM2 function; employing a multi-method approach remains an excellent strategy to overcome the inherent limitations of individual experimental techniques.

In conclusion, the present results demonstrate that TSA improves cardiac function in mice with myocardial infarction and mitigates myocardial damage. TSA ameliorates OGD-induced DC injury by promoting glycolysis and suppressing fatty acid metabolism in DCs. TSA regulates glucose and lipid metabolism through PKM2. Furthermore, TSA may modulate glucose and lipid metabolism in DCs through the dimer of PKM2, thereby influencing DCs’ function. This provides important data for the future clinical application of TSA in the treatment of MI.

## 4. Materials and Methods

### 4.1. Animal Experiment

Male C57BL/6 mice (18–20 g) were purchased from Beijing Huafukang Biological Technology Co., Ltd. (Beijing, China). [license no. SCXK (jing) 2024-0003]. All animal experiments were performed in strict accordance with the National Institutes of Health Guide for the Care and Use of Laboratory Animals and with the approval of the Scientific Investigation Board of Science and Technology of Jilin Province (approval no. 2025-413). The mice were kept in a standard laboratory environment for 7 days. The mice were randomly allocated into six groups, and each group had 15 mice: Sham operation; MI 1 day model; MI 3 day model; MI 7 day model; sham operation + TSA; and MI 3 day model + TSA. The sham operation + TSA and MI 3 day model + TSA groups were treated with 0.2 mg/kg TSA daily by intraperitoneal injection for 10 days; the other groups were injected with an equal volume of saline. MI surgery was performed after injection for 7 days. The procedure was as follows: Mice were anaesthetized using inhalation of 3% isoflurane and maintained under 2% isoflurane with tracheal intubation and mechanical ventilation. The heart was exposed by a left-sided limited thoracotomy, and the left anterior descending coronary artery (LAD) was ligated with 6-0 silk sutures, followed by immediate closure of the thoracic cavity. Mice in the sham surgery group underwent the same procedure without coronary artery ligation. Euthanasia was performed by cervical dislocation on days 1, 3 and 7 after MI in mice. Cardiac tissue and blood samples were collected for subsequent experiments.

### 4.2. Echocardiography

Cardiac function was assessed using echocardiography. The following parameters are used to evaluate cardiac function: left ventricular fractional shortening (FS) and ejection fraction (EF).

### 4.3. 2,3,5-Triphenyl Tetrazolium Chloride (TTC) Staining Experiment

The heart was cut into four equal parts using a blade and incubated in 1% TTC solution. TTC was purchased from Solarbio Science & Technology Co., Ltd. (Beijing, China) at 37 °C in the dark for 5 min. Normal tissue appears red, while the infarct tissue is white. Statistical analysis was performed using Prism 9.0 (GraphPad; Dotmatics; San Diego, CA, USA).

### 4.4. Serum Cardiac Enzyme Testing

Blood was collected from the orbital cavity of mice and centrifuged at 860× *g* for 10 min, and the serum was isolated. Aspartate aminotransferase (AST), creatine kinase (CK), and lactate dehydrogenase (LDH) (GG028423022254255767, XJ007723011631371279, XJ007923011212051557) were purchased from Getein Biotech Inc. (Nanjing, China) and measured according to the manufacturer’s instructions.

### 4.5. Cell Lines and Isolation of Bone Marrow-Derived Dendric Cells (BMDCs)

DC2.4 cells (cat. no. FH0510) were ordered from FuHeng Biology (Shanghai, China) and were authenticated using STR profiling. DC2.4 cells were cultured in RPMI-1640 medium (Gibco; Thermo Fisher Scientific, Inc.; Waltham, MA, USA) supplemented with 10% fetal bovine serum (Gibco; Thermo Fisher Scientific, Inc.) and antibiotics (100 U/mL penicillin and 100 U/mL streptomycin).

Bone marrow (BM) cells were obtained from the femur and tibia of C57BL/6J mice. Red blood cell lysis solution was subsequently added and incubated at room temperature for 5 min. The red blood cells were removed, and the remaining cells were seeded into a 6-well plate with 1 × 10^6^ cells per well. Primary cells were cultured in RPMI-1640 medium, containing 10% fetal bovine serum, 20 ng/mL granulocyte-macrophage colony-stimulating factor (GM-CSF) and 20 ng/mL IL-4 (PeproTech, Inc.; Thermo Fisher Scientific, Inc.; Waltham, MA, USA). Half of the medium was replaced on days 3 and 5. On day 6, the BMDCs were harvested.

### 4.6. Oxygen–Glucose Deprivation Model

Oxygen–glucose deprivation (OGD) model was developed by culturing cells in a hypoxia incubator chamber under 1% O_2_, 5% CO_2_ and 94% N_2_, followed by incubation in glucose-free RPMI-1640 medium (Gibco; Thermo Fisher Scientific, Inc.).

### 4.7. Reagents and Antibodies

TSA (cat. no. HY-15144) and TEPP-46 (cat. no. HY-18657) were provided by MedChemExpress (Monmouth Junction, NJ, USA). Trizol reagent (cat. no. ET111) and the cDNA Synthesis Kit (cat. no. AT301) were provided by TransGen Biotech Co., Ltd. (Beijing, China). The SYBR Green Q-PCR Kit (cat. no. 491391400) was provided by Roche Diagnostics (Basel, Switzerland). The Nuclear-Cytosol Extraction Kit was purchased from Applygen Technologies, Inc. (Beijing, China). The BCA protein assay kit (cat. no. PC0020) was purchased from Beijing Solarbio Science & Technology Co., Ltd. PKM2 (1:1000; cat. no. 4053S) antibody was purchased from Cell Signaling Technology, Inc. (Danvers, MA, USA) β-actin (1:1000; cat. no. YM3028) antibody was purchased from Immunoway Biotechnology Company (Plano, TX, USA). Lamin B1 (1:2000; cat. no. 12987-1-AP), pan acetylation monoclonal antibody (1:1000; cat. no. 66289-1-Ig), CoraLite^®^ Plus 488 Anti-Mouse CD11c (N418) (1:100; cat. no. CL488-65130), goat anti-mouse IgG-HRP (1:5000; cat. no. SA00001-1), and goat anti-rabbit IgG-HRP (1:5000; cat. no. SA00001-2) were purchased from Proteintech Group, Inc. (Wuhan, China) Protein A/G PLUS-Agarose (cat. no. sc-2003) was purchased from Santa Cruz Biotechnology, Inc. (Santa Cruz, CA, USA).

### 4.8. Cell Viability Assay

DC2.4 cell proliferation was assessed using a Calcein/PI Cell Viability/Cytotoxicity Assay Kit (Beyotime Institute of Biotechnology, Shanghai, China). Briefly, DC2.4 cells under OGD conditions were treated with 200 nM TSA for 4 h. Subsequently, 10 μL combined Live/Dead cell-staining solution (2 µM Calcein-AM and 4 µM PI) was added into 500 μL culture medium, followed by incubation at 37 °C for 4 h. Live cells (green fluorescence) and dead cells (red fluorescence) were visualized using an inverted fluorescence microscope.

### 4.9. MTT Assay

After treating DC2.4 cells in different groups, 20 µL 5 mg/mL MTT solution was added to each well. After 2 h incubation, supernatants were discarded and replaced with 200 µL DMSO. Absorbance was measured at 490 nm using a fluorescence microplate reader (Thermo Fisher Scientific, Inc.; Waltham, MA, USA).

### 4.10. Bioinformatics Analysis

The microarray dataset GSE60729 was obtained from the Gene Expression Omnibus (GEO) database (http://www.ncbi.nlm.nih.gov/geo/, accessed on 5 August 2023). These RNA profiles were based on the GPL13667 (HG-U219) Affymetrix Human Genome U219 Array platform. The gene expression differences were compared between three DC (normoxia-non-silenced-unstimulated) samples and three DC (hypoxia-non-silenced-unstimulated) samples. Kyoto Encyclopedia of Genes and Genomes (KEGG) pathway enrichment and Gene Ontology (GO) functional analyzes were performed on GSE60729. GO (http://www.geneontology.org, accessed on 1 September 2023), term enrichment analysis was performed based on biological process (BP), molecular function (MF), and cellular component (CC). KEGG (http://www.genome.jp/kegg, accessed on 10 September 2023), pathway enrichment analysis was performed to identify the signaling pathways associated with the differentially expressed genes. The Benjamini–Hochberg (BH) method was used for multiple-testing correction of the data. Then, the criteria for screening differentially expressed genes were set as adjusted *p* value < 0.05 and |log_2_FC| > 1. For Gene Ontology (GO) and Kyoto Encyclopedia of Genes and Genomes (KEGG) enrichment analyses of differentially expressed genes, we adopted the Benjamini–Hochberg method for multiple-testing correction, with adjusted *p* value (FDR) < 0.05 as the significance threshold.

### 4.11. Reverse Transcription-Quantitative PCR (RT-qPCR) Analysis

Total RNA from DC2.4 cells in each group was extracted using Trizol reagent. cDNA was synthesized from RNA by reverse transcription using a cDNA Synthesis Kit, followed by qPCR with the SYBR Green Q-PCR Kit. Relative mRNA levels were calculated using the Livak method [47] (2^−ΔΔCt^). The PCR protocol began with an initial denaturation at 95 °C for 10 min, followed by 40 cycles of amplification, each consisting of denaturation at 95 °C for 15 s and a combined annealing/extension step at 60 °C for 30 s. All primers were synthesized by Sangon Biotech Co., Ltd. (Shanghai, China), and the primer sequences are listed in Table S1.

### 4.12. Nuclear-Cytosol Separation of DC2.4 Cells and BMDCs

Cells were collected in a 1.5 mL Eppendorf tube and resuspended with cytoplasmic lysis buffer. The mixture was incubated on ice for 30 min, followed by centrifugation to collect the supernatant containing cytoplasmic proteins. The pellet was resuspended with nuclear lysis buffer to extract nucleoproteins, incubated on ice for an additional 30 min and centrifuged to collect supernatant to extract nuclear proteins. Loading buffer was added to the supernatants, which were then boiled for 10 min. Verification of nuclear-cytoplasmic separation purity was performed by collecting equal volumes of protein samples from the nuclear fraction and cytoplasmic fraction. These samples were incubated with specific antibodies against Laminb (nuclear protein marker) and β-actin (cytoplasmic protein marker), followed by Western blot analysis. The detection of a Laminb signal in the nuclear fraction alongside a virtually undetectable β-actin signal, and the detection of a β-actin signal in the cytoplasmic fraction with an undetectable Laminb signal, indicate that the separation purity is adequate for subsequent experiments.

### 4.13. Western Blot Analysis

DCs were lysed with RIPA buffer supplemented with proteinase and phosphatase inhibitors. The protein concentrations were quantified using a BCA protein assay kit. The proteins were separated on 8%, 10% or 12% SDS-PAGE gels and subsequently transferred onto PVDF membranes (Merck KGaA; Billerica, MA, USA). The PVDF membrane was blocked with 5% non-fat milk for 2 h at room temperature, incubated with primary antibodies overnight at 4 °C and then incubated with secondary antibodies. Finally, the PVDF membrane was soaked with ECL reagent (Shanghai ZhongqiaoXinzhou Biotechnology Co., Ltd.; Shanghai, China) for 1 min, and protein bands were developed using an ImageQuant^TM^ LAS 4000 digital imaging system.

### 4.14. Immunofluorescence

Cells were fixed with 4% paraformaldehyde for 30 min, permeabilized with 0.25% Triton X-100 for 30 min, washed with PBS and blocked with 10% BSA for 1 h. Samples were incubated with primary antibody against PKM2 (1:100; Cell Signaling Technology, Inc.; Danvers, MA, USA; cat. no. 4053S) overnight at 4 °C. Goat Anti Rabbit IgG H&L (iFluor™ 488; cat. no. HA1121; Huaan Biotechnology Co., Ltd.; Hangzhou, China) was used as the secondary antibody at a 1:500 dilution and was applied for 1 h at room temperature. Nuclei were counterstained with Hoechst 33342 (1:1000; Thermo Fisher Scientific, Inc.; Waltham, MA, USA). Immunofluorescence images were acquired using fluorescence microscopy (Zeiss AG, Oberkochen, Germany).

### 4.15. Lactate, Oxaloacetate, Fatty Acid and Acetyl Coenzyme A Assay

A Free fatty acid (FFA) Content Detection Kit (cat. no. BC0595; Beijing Solarbio Science & Technology Co., Ltd.), Acetyl coenzyme A Content Detection Kit (cat. no. BC0980; Beijing Solarbio Science & Technology Co., Ltd.), Oxaloacetate Content Detection Kit (cat. no. YX-150100M; Youxuan Biotechnology Co., Ltd.; Shanghai, China) and Lactic acid Content Detection Kit (cat. no. BC2235; Beijing Solarbio Science & Technology Co., Ltd.) were used to assess the corresponding contents according to the manufacturer’s protocols.

### 4.16. Cell Transfections

Small interfering (si)RNAs were transfected into DC2.4 cells using Lipofectamine 2000 (Invitrogen; Thermo Fisher Scientific, Inc.; Waltham, MA, USA). Si-PKM2 was obtained from Guangzhou RiboBio Co., Ltd. (Guangzhou, China). siRNA sequences were as follows: si-PKM2 (5′-GCAAGAACATCAAGATCAT-3′).

### 4.17. Disuccinimidyl Suberate (DSS) Crosslinking

Crosslinking was performed according to the protocol of the DSS crosslinker agent (Thermo Fisher Scientific, Inc.) [48]. Initially, an equal number of cells were collected for each group and washed twice with PBS. A DSS solution prepared in DMSO was added to a cell suspension at a final concentration of 1 mM, and subsequently incubated at room temperature for 30 min. The quenching solution (10 mM Tris-HCl) was added, and the samples were incubated at room temperature for 15 min. The sample was subsequently boiled for Western blot analysis.

### 4.18. Acetylation Assay

Cellular proteins were collected and incubated overnight at 4 °C with the PKM2 antibody. Subsequently, the mixtures were incubated with the Protein A/G PLUS-Agarose beads for 2 h at 4 °C. The beads were washed with lysis buffer followed by elution with loading buffer. The eluted proteins were analyzed using Western blotting, and an acetylation antibody was used to detect PKM2 acetylation.

### 4.19. Flow Cytometric Analysis

The mouse heart was washed with PBS to remove blood and subsequently cut into pieces and incubated in 2 mg/mL collagenase II (Beijing Solarbio Science & Technology Co., Ltd.; cat. no. C8150) at 37 °C with shaking at 210× *g* for 45 min to digest the cells. The resulting solution was filtered through a 70 μm filter and centrifuged at 100× *g* for 5 min. The supernatant was discarded, and 5 mL red blood cell lysis buffer (Beyotime Institute of Biotechnology; cat. no. C3702) was added to each tube. The mixture was thoroughly mixed and allowed to sit for 5 min to ensure complete lysis of the red blood cells. Afterwards, the mixture was centrifuged again at 100× *g* for 5 min, and the supernatant was discarded to remove the lysed red blood cells. The remaining cells were harvested and stained with anti-mouse CD16/32 (1:100; Beyotime Institute of Biotechnology; cat. no. C1755S) at 4 °C for 10 min. Subsequently, the cells were stained with extracellular antibodies on ice for 30 min, including BV421-anti-CD45 (1:100; Proteintech Group, Inc.; cat. no. CL405-98035), APC-anti-CD11c (1:100; eBioscience; Thermo Fisher Scientific, Inc, cat. no. 17-0114-81) and PE-anti-MHC II (1:100; eBioscience; Thermo Fisher Scientific, Inc.; cat. no. 12-5321-82). Following this, the cells were fixed (Beyotime Institute of Biotechnology; cat. no. C1713) and permeabilized (Beyotime Institute of Biotechnology; cat. no. C1715). Then, the intracellular antibody FITC-anti-PKM2 (1:100; Proteintech Group, Inc.; cat. no. CL488-60268) was used to stain the cells at room temperature in the dark for 30 min. Subsequently, the cells were washed with PBS and analyzed using a BD FACS Aria II. Data analysis was performed with FlowJo V10.0 software (BD Biosciences; San Jose, CA, USA).

### 4.20. Statistical Analysis

Data are expressed as the mean ± standard deviation (SD). An unpaired two-tailed Student’s *t*-test was used for comparisons of two groups. One-way analysis of variance (ANOVA) was used for comparisons among multiple groups, followed by Tukey’s multiple comparisons post hoc test. Data were analyzed using GraphPad Prism 9 software. All experiments were independently repeated at least three times. *p* < 0.05 was considered to indicate a statistically significant difference. All experiments were performed with biological replicates.

## Data Availability

The data generated in the present study may be requested from the corresponding author.

## Supplementary Materials

The following supporting information can be downloaded at: https://www.mdpi.com/article/10.3390/molecules31020319/s1. Figure S1: Generation of BMDC; Figure S2: Interference effect detection after siPKM2 interference with DC2.4; Table S1: Primer sequences used for RT-qPCR.
