# Supplementary material for: Trichostatin A Influences Dendritic Cells’ Functions by Regulating Glucose and Lipid Metabolism via PKM2
Source: Molecules. 2026 Jan 16;31(2):319. doi: 10.3390/molecules31020319 (PMC12844180; doi:10.3390/molecules31020319)
Supplement: Supplementary file 1 [file molecules-31-00319-s001.zip › molecules-4079307-supplementary/Figure S1, S2.pdf]

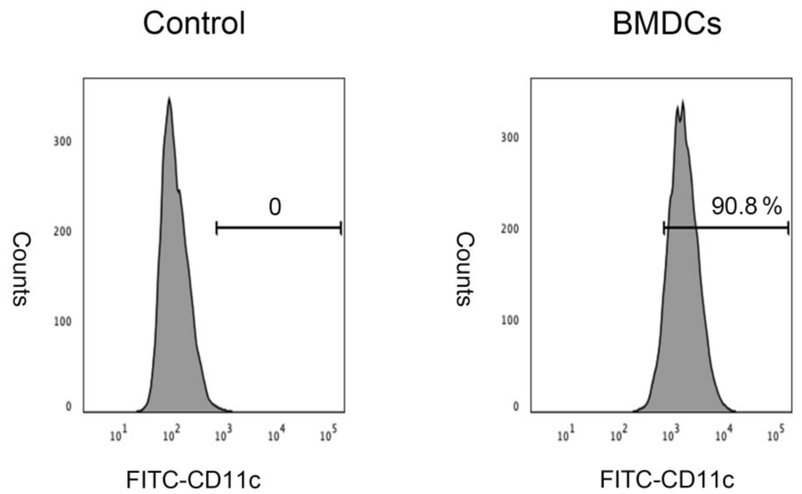

Figure S1. Generation of BMDC.BMs were obtained from mouse femurs and tibias and treated with GM-CSF (20 ng/mL) and IL-4 (20 ng/mL) for 6 days. The purity of CD11c+ BMDCs was detected by flow cytometry.

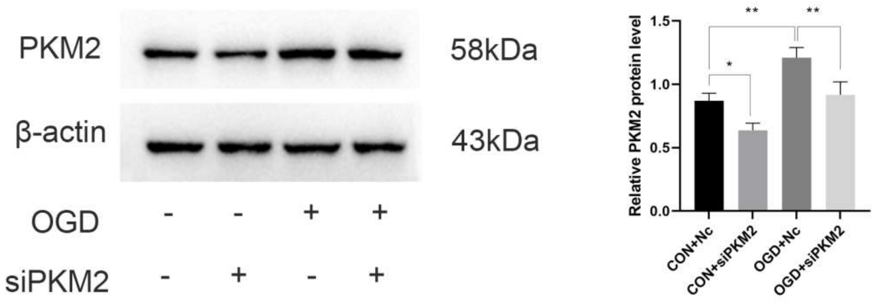

Figure S2. Interference effect detection after siPKM2 interference with DC2.4. Interference effect of PKM2 was detected by western blot after siPKM2 interference with DC2.4 cells for 48h. * $p < 0.05$, ** $p < 0.01$.
