# Supplementary material for: Trichostatin A Influences Dendritic Cells’ Functions by Regulating Glucose and Lipid Metabolism via PKM2
Source: Molecules. 2026 Jan 16;31(2):319. doi: 10.3390/molecules31020319 (PMC12844180; doi:10.3390/molecules31020319)
Supplement: Supplementary file 1 [file molecules-31-00319-s001.zip › molecules-4079307-supplementary/Table S1.pdf]

Table S1. Primer sequences used for RT-qPCR

Gene	Forward (5′-3′)	Reverse (5′-3′)
Hk2	TGATCGCCTGCTTATTCACGG	AACCGCCTAGAAATCTCCAGA
Ldha	TGTCTCCAGCAAAGACTACTGT	GACTGTACTTGACAATGTTGGGA
Pdk1	GGACTTCGGGTCAGTGAATGC	TCCTGAGAAGATTGTCGGGGA
Glut1(Slc2a1)	TCAAACATGGAACCACCGCTA	AAGAGGCCGACAGAGAAGGAA
Pfkfb3	CAACTCCCCAACCGTGATTGT	TGAGGTAGCGAGTCAGCTTCT
Pkm2	GTCTGGAGAAACAGCCAAGG	CGGAGTTCCTCGAATAGCTG
Ogdh	GTTTCTTCAAACGTGGGGTTCT	GCATGATTCCAGGGGTCTCAAA
Cs	GGACAATTTTCCAACCAATCTGC	TCGGTTCATTCCCTCTGCATA
Idh2	ATTTTGTGGTAGATCGAGCTGG	CCTCCGGCAGGGAAGTTATAC
Mdh2	TGTTCAACACCAACGCTACCA	AAGGGTTGTCACACCGAAGAT
Acaca	AATGAACGTGCAATCCGATTTG	ACTCCACATTTGCGTAATTGTTG
Fasn	GGAGGTGGTGATAGCCGGTAT	TGGGTAATCCATAGAGCCCAG
Srebp-1(srebf1)	CTTTGGCCTCGCTTTTCGG	TGGGTCCAATTAGAGCCATCTC
Acly	CAGCCAAGGCAATTTCAGAGC	CTCGACGTTTGATTAACTGGTCT
Acs(Acss2)	GTGGATGAAAGGAGCAACTACA	GCCCTCCCAGTAAAAAGCAACT
Scd1	TTCTTGCGATACACTCTGGTGC	CGGGATTGAATGTTCTTGTCGT
Cpt1a	CTCCGCCTGAGCCATGAAG	CACCAGTGATGATGCCATTCT
Cpt2	CCTGCTCGCTCAGGATAAACA	GTGTCTTCAGAAACCGCACTG
Acadm	AGGGTTTAGTTTTGAGTTGACGG	CCCCGCTTTTGTCATATTCCG
Acads	TGGCGACGGTTACACACTG	GTAGGCCAGGTAATCCAAGCC
Hadh	TCAAGCATGTGACCGTCATCG	TGGATTTTGCCAGGATGTCTTC
Hadhb	ACTACATCAAAATGGGCTCTCAG	AGCAGAAATGGAATGCGGACC
Hdac2	GGAGGAGGCTACACAATCCG	TCTGGAGTGTTCTGGTTTGTCA
Hdac1	GACGACGAATCCTATGAAGCCATC	GGAATCTGAGCCACACTGTAAGAC
Hdac3	AGCCAGTCATCAGCCAGGTG	CGATCACAGCCCAGGGAGTC
Hdac4	CTGGCATCCCTGTGTCATTTGG	CTGTGGTGAACCTTGGCTTGG
Hdac5	GAAGCAGCACGACCACTTGAC	TCTTAGCCGCCAGCATCTCC

Hdac6	AGGAAGACCATAGCAGCACTACC	GCAAGCACAGCCTTAGCCATC
Hdac7	TCCGCAGCCAGTGTGAGTG	TGAGTGGGTTCGTGCCGTAG
Hdac8	GACGGGAAGTGTAAAGTAGCCATC	CCAGGACAGCATCATTGAGATAACAG
Hdac9	GATGATGATGCCTGTGGTGGATC	ATCTGCTGCTGCTGCTGAATAAG
Hdac10	TCTGCGTCTGCTTGGTGAGAG	GGGCGGAGGCACAGTTGG
β-actin(Actb)	TAGGCGGACTGTTACTGAGC	TTTGGGGGATGTTTGCTCCA
